# Social Investment after Neoliberalism: Policy Paradigms and Political Platforms

**DOI:** 10.1017/S0047279414000828

**Published:** 2015-04

**Authors:** CHRISTOPHER DEEMING, PAUL SMYTH

**Affiliations:** * School of Geographical Sciences, University of Bristol, UK email: chris.deeming@bristol.ac.uk; ** School of Social and Political Sciences, University of Melbourne, Australia email: p.smyth@unimelb.edu.au

## Abstract

The concept of the ‘social investment state’ refocuses attention on the productive function of social policy eclipsed for some time by the emphasis on its social protection or compensation roles. Here we distinguish between different social investment strategies, the Nordic ‘heavy’ and the Liberal ‘light’, with particular reference to the inclusive growth approach adopted in Australia. In 2007, social democrats in Australia returned to government with a clear mandate to reject the labour market deregulation and other neoliberal policies of its predecessor, and to tackle entrenched social and economic disadvantage in Australian society. For the last five years, social investment and inclusive growth has been at the centre of the Australian social policy agenda. Against this background, the article examines and critically assesses the (re)turn to ‘social investment’ thinking in Australia during Labor's term in office (2007–13). Analysis focuses not just on what was actually achieved, but also on the constraining role of prevailing economic and political circumstances and on the processes that were used to drive social investment reform. In many ways, the article goes some way to exposing ongoing tensions surrounding the distinctiveness of ‘social investment’ strategies pursued by leftist parties within the (neo)liberal state.

## Introduction

In this article, we critically examine the concept of ‘social investment’ and the political strategies pursued by social democratic parties in power. Here we distinguish between investment platforms, the Nordic ‘heavy’ and the Liberal ‘light’, with particular reference to the inclusive growth strategy adopted by the Australian Labor Party during its term in office (2007–13). We critically examine the reasons for Labor adopting a ‘social investment’ approach. In so doing, the article provides a critical analysis of the content and coherence of Labor's social investment strategy in terms of policy change (cf. Hall, 1993). Analysis focuses not just on what was actually achieved, but also on the constraining role of prevailing economic and political circumstances and on the processes that were used to drive social investment reform. More broadly, we are interested in situating the Australian case within the growing literature on ‘social investment’, and we contrast the Australian ‘light’ approach with the ‘heavy’ investment strategies pursued elsewhere in Northern Europe. In many ways, the article goes some way to exposing ongoing tensions surrounding the distinctiveness of ‘social investment’ strategies pursued by leftist parties within the (neo)liberal state.

The concept of a ‘social investment state’ has a deep resonance with an Australian social policy history strongly characterised by productivist values and giving each other a ‘Fair Go’ (opportunity). We illustrate this briefly from the classic moments in the evolution of the ‘Australian Way’ before reflecting on the (re)turn to ‘social investment’ policies in the early part of the twenty-first century. We show how this productivist revival helped break the austerity constraints on Australian social policy created in the neoliberal period. At the same time, we find that the contemporary concept of ‘the social investment state’ is only a partial expression of this productivist tradition and remains politically fragile because of deep uncertainties around the future of domestic economic policy. We also find that, because of its rather unique history and policy pathway of achieving redistributive efforts using the instruments of wage regulation (rather than public expenditure in traditional areas of welfare), Australia has a particular challenge of reviving the productive dimension of social policy in a way that is not detrimental to its protective or compensatory functions. Needless-to-say, some of the longer-term outcomes and returns on investments in areas such as early childhood development, mental health, education and social mobility, for example, cannot be accommodated in this review.

## Social investment: emerging concept and policy context

‘Social investment’ arguably represents the very latest justification for social policy to guide the development of the economy and society in the twenty-first century. The early origins of the social investment perspective are traceable to the founding of the Swedish social-democratic welfare state in the 1930s, and the arguments of Swedish social democrats who viewed social policy as an *investment* rather than a cost (Tilton, 1993). Since the late 1990s, new ideas and strategies concerning the role of social policy for societal development have been formulated, and, internationally, policy agendas now point towards a similar policy logic based around notions of ‘social investment’ (Midgley, 1999). The notion of ‘social investment’ makes reference to policies that aim to help disadvantaged citizens to succeed in education and the labour market. As Jenson (2012) argues, ‘social investment’ can be distinguished both from the more traditional ‘Keynesian’ social policy approach based on income and job protection and from the ‘neoliberal’ one based on deregulation and the commodification of human labour. Nevertheless, social policy scholars do not share a singular theoretical core on a par with macro demand-side management or supply-side economic liberalism, nor could we say that the ‘social investment’ perspective has fully emerged as the dominant policy paradigm (see, for example, Hemerijck, 2012). Despite some of the conceptual vagueness, two core features may be observed: investment in human capital and the objective of full labour market participation. Indeed, the notion that the social investment state seeks to rebuild the welfare state around work has become iconic in the European context but, as we shall see, this has been a long-standing tradition in the Australian context. Conceptually, scholars attempt to separate out ‘social investment’ spending from social spending on ‘old social risks’ relating to pensions, healthcare and unemployment benefits (but this can be problematic as we shall see). More broadly, scholarship continues to observe distinctions between different ‘worlds’ (Morel *et al.*, 2012) and ‘varieties’ of social investment regime (Bonoli, 2009), with further distinctions between different investment strategies. These may be described as ‘strong’ or ‘weak’ for example (Bonoli, 2012), or (as we prefer) ‘heavy’ and ‘light’, and our notion of ‘Social Policy as Productive Factor’ may go beyond that originally envisaged in Europe in the late 1990s (European Commission, 2000).

**Figure 1.**
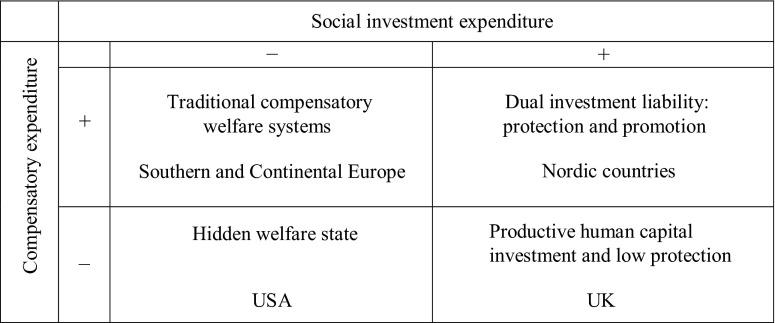
Four worlds of social investment *Source:* Adapted from Morel *et al.* (2012: 358).

The experience of the Nordic countries suggests that it may be perfectly possible to combine strong social protection (i.e., ‘old’ social spending) with heavy investment in human capital (i.e., ‘new’ social spending, particularly in education and early childhood policies) in order to secure greater levels of equality and foster the human capital of future generations (Kvist 2014). According to Esping-Andersen *et al.* (2002) social policy should actively mobilise the productive potential of citizens in order to mitigate the ‘new’ social risks of the post-industrial knowledge-based economy (adversely affecting low-skilled workers, women, young adults and children). New family- and child-centred investment strategies, for example, can break patterns of social inheritance and exclusion. Social protection, however, is of paramount importance even in the most productivist of welfare states, as Esping-Andersen (2001) argues. The human capital-based investment strategy will inevitably leave some citizens behind, and, thus, it remains an imperative to have a secure welfare safety net of minimum income support underpinning the social investment strategy. Social democrats in market liberal economies such as Labour (between 1997 and 2010) in the UK, by contrast, have pursued lighter social and human capital-based investment strategies alongside the pro-market employment orientation of welfare provision. Here ‘passive’ welfare systems, which had created a ‘moral hazard’, were transformed by ‘active’ social policies.^1^ The role of the state was to facilitate ‘activation’, with new training programmes to improve skills, and new forms of welfare ‘conditionality’ to tackle the wider cultural problem of ‘worklessness’ in society. Tax and benefit reforms, and incentives, helped to ‘make work pay’. This policy platform was inspired by ‘Third Way’ social democratic thinking advocated by Anthony Giddens (1998), and later embraced by the European Commission and the OECD (Hemerijck, 2012; Jenson, 2010). In Figure 1, we see the different investment strategies which show the Nordic dual investment approach (i.e., protection and promotion), and the UK's human capital investment approach combined with low social protection. The USA is taken as an example of a weakly developed social investment state.

The policy context in Australia, however, is very different to that found in Europe. Here we find the emergence and foundations of Australia's ‘social investment state’ at the end of the nineteenth century and the beginning of the twentieth century (this has been well documented elsewhere, Perkins *et al.*, 2005; Smyth, 2008). Its novelty lay in the emphasis on the role of social policy as economic investment. Thus, social investment played a key role in the economy, with extensive government investment in capital infrastructure, utilities and state enterprises. Industrial regulation provided a formative social investment pathway and the orientation towards a ‘welfare society’, rather than a ‘welfare state’. Minimum wage laws were introduced in South Australia in 1894 and then, in 1907, the ‘Harvester Judgment’ gave courts in Australia the power to determine acceptable working conditions including (minimum) living wages for families. Thus, a social economy with a fair wage was the order of the day. Clearly, an inclusive growth strategy has always been at the heart of the ‘Australian Way’. Even the so-called ‘wage earners’ welfare state’ only made sense – and in fact only ever worked – when integrated within a successful growth strategy designed to deliver more and better-paid manufacturing jobs in the industrial period.

Across the 1980s and 1990s, social policy had progressively reduced its role to social protection or compensation, vacating the sphere of economic production to the newly revived free market economics. Welfare slowly shrank to the dimension of an income ‘safety net’, and thus seemingly faced a future of permanent austerity. However, with the election of the Rudd Labor government in 2007, we observe a return to the characteristically Australian productivist tradition of active state investment. The ‘social investment’ agenda emerged first as an economic policy agenda aimed at lifting the performance of the nation's ‘human capital’ (especially disadvantaged groups who were measurably underachieving). This was overlaid by a ‘social inclusion’ agenda belatedly adopted from Europe. As we shall show below, social policies were soon being politically reframed in the languages of ‘investment’ and ‘inclusion’, although the narratives lacked depth and the policies any rigorous framework of the means for and goals of social investment (Saunders, 2013).

In his critique of ‘social investment’, Nolan (2013) distinguishes the ‘paradigm’ from the ‘platform’. We shall show that social investment is already embedded as a platform in Australia. But we also explore what the Australian experience can tell us about it as a paradigm. Here we have three main points of departure. The first arises from the observation that the agenda requires a more integrated approach to economic and social policy than that which was obtained for much of the postwar period (Smyth and Buchanan, 2013). Especially after the triumph of neoliberal economics in the 1980s and 1990s, social policy found itself subordinated to the market with little room to construct a positive role for state intervention. Thus, when economic policy actually shifted to allow space for a social investment agenda which would tackle the negative effects of poverty and inequality, even while promoting economic growth, many in social policy remained suspicious of an ongoing subordination of the social to the economic (Nolan, 2013).

But the challenge to a better integration of economic and social policy runs more deeply through the discipline. Thus, Beblavý *et al.* (2013) describe the difficulties in integrating a study of education with social protection in the OECD, when the first is positioned as being about ‘opportunities’ and the second about ‘outcomes’. Education of course is the vanguard of the social investment approach. The narrow preoccupation of the social policy tradition with the tax and transfer system has also stifled dialogue with development policy where the inclusive growth approach offers a more complete integration of the economic and the social than that so far afforded by the social investment framework. Midgley (1999), for example, writes of social policy's inability to take full account of the role of social policy in growth and structural change. To progress ‘social investment’, the new role of social policy cannot just be about protection but about building the productive capacities of citizens – principally, but not exclusively, their education and health.

This leads to our second challenge. While the positive social policy environment in Australia has partly reflected an economy that has avoided recession for over two decades, today's global economic environment highlights that a social investment platform by itself is not enough if the economy does not deliver sufficient employment opportunities. Here social policy cannot be indifferent to the different implications for its role of either a persistent neoliberal ‘growth first’ strategy or a shift to the alternative of ‘inclusive growth’ currently being developed by the OECD and Bretton Woods institutions. In the inclusive growth strategy, government seeks to influence the pattern of economic development to ensure opportunities for all citizens (OECD, 2014). The third challenge – and one very much a focus of this paper – is already well canvassed in the literature: that is the tendency for the productivist values of social investment to be promoted at the expense of, rather than as a complement to, the value of social protection. Here the Australian case is of particular interest because we show how the recent renewal of the characteristically productivist welfare tradition has indeed been accompanied by greater constraints around aspects of its income support system.

Australia is an interesting site on which to explore these issues. The neoliberal period brought radical not incremental change to the traditional Australian welfare state model. In the mid-1990s, the Australian welfare regime was well recognised in the international literature as a fourth – ‘radically redistributive’ – world, an exception to Esping-Andersen's threefold typology. Australia became routinely identified as the ‘wage earners’ welfare state’ (see Deeming, 2013a, for a critical review of this literature). By the close of the twentieth century, this model had been largely confined to history and the new social investment state was being designed around the needs of ‘hardworking’ Australian families.

## Australia's (re)turn to social investment

If the oil crises of the 1970s helped to usher in the neoliberal chapter in Australian politics, then the global financial crisis helped to instigate a return to social investment (Saunders and Deeming, 2011). The Rudd Labor government, which had been elected in 2007, reverted to ‘Keynesian’ social investment policies in order to stimulate the economy in the face of recession (showing the interchange of economic ideas, see Hall, 1989, on different variants of Keynesianism). ‘Nation Building’, rather than neoliberal austerity measures, point to the productive interpretation and value of social interventions as ‘social policy’ under Labor. Capital spending on national infrastructure projects and new investment in road, rail and port infrastructure, for example, may be outside the more narrow confines of the list of policy proposals contained in the Social Investment Package produced by the European Commission (2013), but we might well question the logic of this framework. Such questioning of the term and of what might reasonably be included or excluded under a ‘social investment strategy’ is a recurring theme in this article.

Labor politicians now distanced themselves from neoliberal ideology, arguing instead for social democratic values and principles of social investment and inclusion to help make Australia a fairer society. As we shall see, however, there were strong continuities but also critical differences from the ‘neoliberal’ agenda, particularly with the role of the ‘enabling’ or ‘active’ investment state taking greater responsibility for workforce participation and for skills, training and employment strategies, as articulated by social democrats in Europe (Esping-Andersen *et al.*, 2002). Keynesian principles of social investment in human capital and infrastructure were also revived under Labor. The traditional social democratic concern with social justice also remained, but the relationship between the state and recipients of welfare was now recast in a new contract of ‘rights and responsibilities’, framed by new thinking about inclusive economic growth (Smyth and Buchanan, 2013). The Labor government now firmly believed in social inclusion through paid work. Policymakers agreed that all working-aged adults who could work should be engaged in the labour force, thus increasing the centrality of paid work to the securing and production of family well-being in Australia.

In the lead-up to the 2007 election, Labor had announced its support for a ‘social inclusion’ policy. The approach rested on the assumption that a purely income-based approach to disadvantage does little to address the underlying causes of social disadvantage. Social inclusion initiatives focused on social and economic participation as a means of overcoming social disadvantage. The social inclusion agenda had two guiding principles: firstly, it must tackle the social exclusion of individuals and communities; and, secondly, it must invest in the human capital of citizens, especially the most disadvantaged. The goal for Labor was to reduce state spending on unproductive areas of social welfare, such as social security benefits, by increasing labour market participation and inclusion. Access to paid work was not only seen as the best welfare guarantee, but higher rates of labour market participation also increases the tax base and thus helps to sustain other welfare state functions, such as healthcare and pensions for older citizens in retirement.

**Figure 2.**
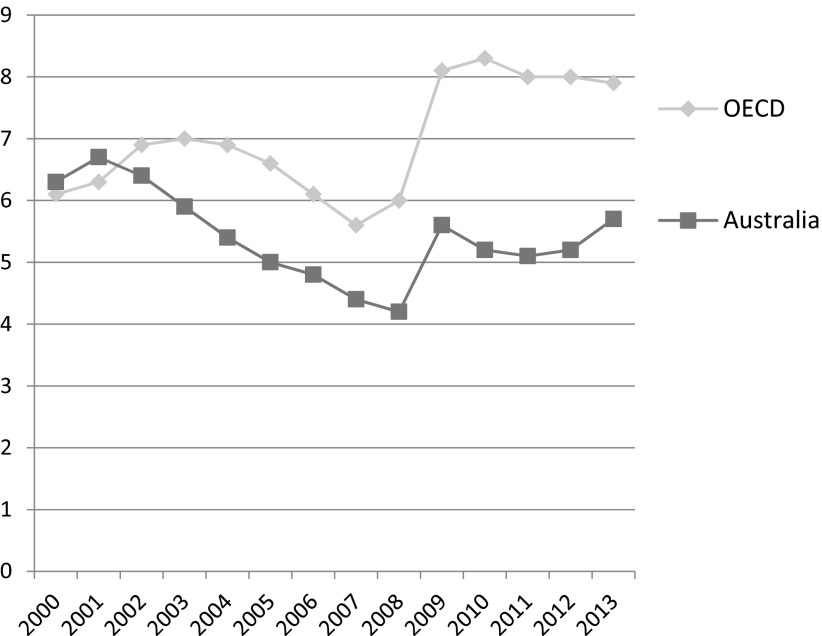
Unemployment rates (all adults) in the advanced economies, 2000–13 *Source:* OECD Employment and Labour Markets Statistics (DOI: 10.1787/lfs-data-en).

The Labor government regarded work as the best route out of poverty for unemployed Australians, although it was now content to pursue the (diminished) goal of full employability, as opposed to full employment. Australia has one of the lowest unemployment rates among the advanced economies (Figure 2), and the number of people out of work has risen following the economic downturn in 2008.

Nevertheless, the legitimate role of the active welfare state was to mobilise the full productive potential of Australian citizens. New ‘activation’ and job training programmes helped to reskill workers and increase their job readiness. Long-term unemployment can deskill in the knowledge economy. Training unemployed citizens in the skills required by employers was therefore part of the supply-side approach adopted by the Labor government to overcome shortfalls in the economy. Compared to European countries, however, the level of state investment in human capital policies and active labour market programmes (ALMPs) in Australia remained relatively low by comparison, and their effectiveness to generate successful employment outcomes in the Australian context is questionable (Belchamber, 2013). Table 1 shows total annual expenditure on active labour market policies in Australia at 0.3 per cent of GDP for the years 2007–11. Denmark had the highest expenditure on ALMPs at 2.3 per cent of GDP in 2011. The relatively low level of investment in ALMPs may surprise, given Labor's firm commitment to social investment and inclusion. However, if social investment in ‘human capital policies’ is more broadly conceived to include education (including vocational education and training in schools and universities), we find Australia has above average spending and performs well on educational and training outcomes (Burke, 2013). There is widespread consensus that advanced welfare states have to maintain a highly skilled and highly productive workforce in order to maintain living standards and competitive advantage in the global economy.

**TABLE 1.** Public expenditure on active labour market policies in the advanced economies (% of GDP)

	2004	2005	2006	2007	2008	2009	2010	2011
Denmark	1.8	1.6	1.6	1.3	1.4	1.7	2.1	2.3
Sweden	1.1	1.2	1.2	1.0	0.9	0.9	1.1	1.1
Finland	1.0	0.9	0.9	0.9	0.8	0.9	1.1	1.0
France	1.0	0.9	0.9	0.9	0.9	1.0	1.1	0.9
Germany	1.1	1.0	0.9	0.7	0.8	1.0	0.9	0.8
Norway	0.8	0.7	0.6	0.6	−	−	−	−
UK	0.5	0.4	0.3	0.3	0.3	0.4	−	−
Australia	0.4	0.4	0.3	0.3	0.3	0.3	0.3	0.3
New Zealand	0.4	0.4	0.4	0.3	0.4	0.4	0.3	0.3
United States	0.1	0.1	0.1	0.1	0.2	0.2	0.1	0.1

*Source*: OECD Employment and Labour Markets Statistics (DOI: 10.1787/lfs-data-en).

**Figure 3.**
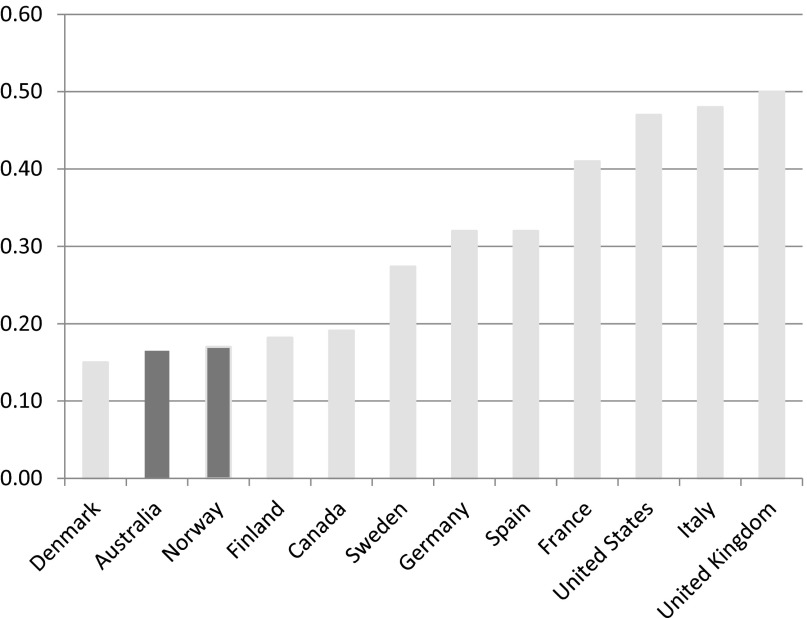
The strength of the link between individual and parental earnings in the advanced economies *Note:* The height of each bar measures the extent to which sons’ earnings levels reflect those of their fathers. The higher the value, the greater is the persistence of earnings across generations, thus the lower is the intergenerational earnings mobility. *Source:* OECD (2010: 181–98).

The Labor government embraced the latest social investment principles that prioritise high levels of employment for both men and women. New family- and child-centred social policies were introduced that effectively ‘hollowed out’ the old ‘male bread-winner/female home-maker’ (or ‘wage earner’) model of welfare in favour of the new dual-earner model of family-centred welfare production (Deeming, 2013a). In terms of policy redirection, this welfare effort looked to privilege the active stages of the life course, but here Labor was also attempting to promote social mobility. For Labor's policy of social inclusion also committed the government to tackling social exclusion at birth. Comparatively, Australia performs well on most indicators of intergenerational social mobility, which is concerned with the relationship between the socio-economic status of parents and the status their children will attain as adults. Figure 3, for example, shows the strength of the link between the individual and parental earnings. In Australia, this link is relatively low compared to the UK, where it is relatively high.

Labor focused much of its efforts on strengthening the system of welfare for ‘hardworking families’; this term, which has positive connotations, was extensively used by Rudd and Gillard during the 2007 federal election. Successive Labor budgets (2008–12) were mildly progressive, intent on improving the living standards for this group. The government provided tax relief for low-income households, the Low Income Tax Offset, and child-care tax rebates to the tune of $47 billion, such as the Child Care Rebate and Family Tax Benefit, to help provide for their needs. The Labor administration also introduced the national Paid Parental Leave scheme in 2011. Thus, a new model of employment relations is in the making whereby both men and women share both working and family time.

The very notion of ‘hardworking Australian families’, the preferred expression of Prime Minister Gillard in her 2010 Address to the Nation, is not just a rhetorical device aimed at a political constituency – it also articulates a range of normative assumptions about the way in which life should be lived in Australia in the twenty-first century. Work is seen as the best form of welfare for Australian families, not only because work provides greater financial rewards, but also because it promotes individual and social well-being. Therefore, regulatory work-based welfare measures that had been introduced in the 1990s were strengthened in order to help move welfare claimants into paid employment. Thus, increased benefit conditionality was perfectly justifiable in order to tackle what policymakers saw as the growing social problem of ‘welfare dependency’ in Australia and the culture of ‘worklessness’ that had evolved out of the passive system of social security transfers. With the emphasis on individual responsibility, reciprocity and obligation, there are strong continuities here with neoliberal workfare-orientated policies, as benefit claimants, particularly parents with young children, now found new conditions and forms of enforcement attached to social security payments: being available for work and actively seeking employment and being able to demonstrate this, for example, were minimum codes of conduct. Harsh punitive sanctions help to guard against non-compliance (Blaxland, 2013).

Labor's Fair Work Act reversed the unpopular labour market polices of the 1990s and improved job security for workers. In particular, Labor restored to Australian workers the legal right to appeal against harsh or unfair dismissals from their place of work – a right that had been rescinded by the Workplace Relations Amendment Act 2005, known as WorkChoices. Once again, collective bargaining was encouraged and wages were to reflect relative living standards and the needs of workers (as well as the potential impact of changes on the labour market and unemployment levels). Low-paid workers clearly benefited. Despite the falling value of minimum wages during the 1980s and 1990s, minimum wage protection levels in Australia increased during the 2000s, offering low-paid workers better protection from the vagaries of the market.

Targeted tax cuts for working families and the Fair Work initiative, which reinstated the notion of appropriate minimum wage rates on social justice grounds, clearly marked a return to more familiar territory for Australian social policy. However, the Australian ‘social investment’ discourse was clearly not just about protecting low-income working families from the perils of the free market. Labor had embraced the latest social investment principles with the new family- and child-centred policies, child-care rebates and tax breaks that now saw more affluent ‘middle-class’ families drawn into the ‘fiscal’ welfare system, thus creating a ‘dual’ welfare system in Australia.

Significantly, this ‘social investment state’ reform agenda in Australia emerged from economic not social policy – perhaps explaining why social policy research has been so slow to recognise the major welfare reform trend it embodies. Also it originated under the Liberal–National coalition government led by John Howard (1996–2007), underscoring its bipartisan appeal. The catalyst was the call for a ‘third wave of national reform’ by the Victorian state government (Australian Department of the Premier and Cabinet, 2005). It described the success of the first two waves in reinvigorating a national economy after significant decline in the 1980s. Thus, the first wave opened up the economy in that decade principally through tariff reductions and financial deregulation, while the second focused on microeconomic reform and was driven through the National Competition Policy framework established in 1995. While emphasising the ongoing value of these reforms, the report insisted that participation and productivity had now emerged as the critical drivers of future prosperity. ‘The most effective way’, it said, to boost productivity and participation is to develop our human capital. Improving health, learning and work outcomes is how we build a healthy, skilled and motivated society, and a high income economy that is among the world's best’ (Australian Department of the Premier and Cabinet, 2005: 8).

The Council of Australian Governments’ (COAG) Reform Council was established in 2006 and a national reform agenda commenced in 2008. Chairman John Brumby (2013) summarised the work after five years in a way which shows the coalescence of economic and social goals. ‘There were two main elements’, he said, ‘further regulation reform, and investment in human capital . . . (it) was all about improving the lives of Australians – their health and well-being, their skill levels and education, their participation in work and in society more broadly . . . It would be good for individuals, good for families, but also good for an economy whose greatest future strength would lie in the resilience, creativity and capacities of its people’. The key social policy areas related to the early years, schooling, the employment participation of youth and at-risk groups; better health services and systems; and closing the gap on indigenous disadvantage. It is a policy profile which matches broadly that associated with the Social Investment Package in Europe – relating to the early years, youth, activation and health (European Commission, 2013).

While often referred to as the ‘human capital’ agenda, the breadth of the COAG reforms points to the influence on Australian policymakers of Sen's work on capabilities (Sen, 2005), and the capabilities approach has also underpinned the Federal Treasury's economic well-being framework. This approach, said COAG's ‘Third Wave’ report, ‘considers not only incomes, but also health and education outcomes . . . A human capital approach therefore supports not only economic outcomes, but also the public interest more broadly’ (Australian Department of the Premier and Cabinet, 2005). The COAG Reform Council's five-year progress review found that the implementation and outcomes had been a mixed success. While endorsing the overall policy agenda, it noted that there was some loss of reform momentum as a result of the global financial crises, the changing political composition of member governments and the general challenges created to national reform agendas by Australia's federal system of government. Further, many significant areas of reform relating to human capital have subsequently occurred outside of the COAG framework. Whether or not COAG continues as the vehicle of national reform in Australia remains to be seen. However the policy directions which it has set have provided Australia with the base for that transition from a welfare to a social investment state integral to the inclusive growth approach (Smyth and Buchanan, 2013).

**Figure 4.**
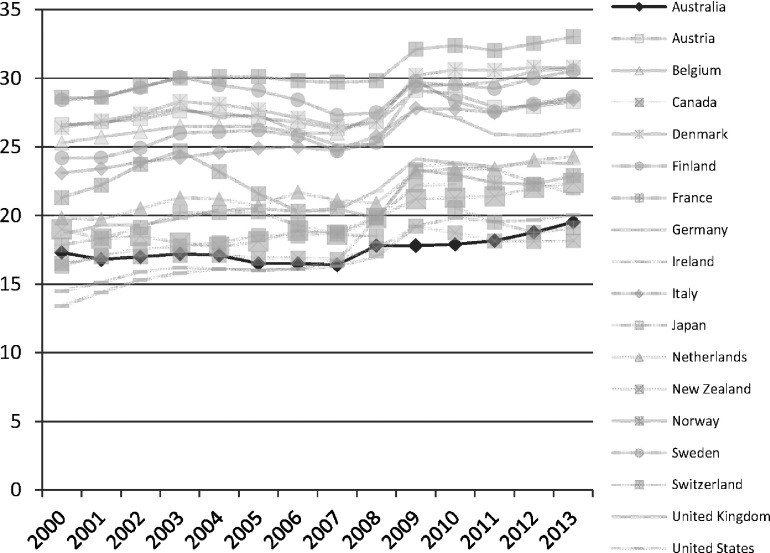
Total public social expenditure in the advanced economies, 2000–13 (as a percentage of gross domestic product) *Source:* OECD Social Expenditure Statistics (SOCX data, DOI: 10.1787/socx-data-en).

The Labor administration also secured major investment in ‘old’ social welfare protection against social risks during its term in office, securing real improvements to the standard of living for pensioners and people with a disability, although the extent to which all forms of ‘social spending’ may be differentiated between ‘social investment’ spending (looking for ‘returns’) and ‘non-social investment’ spending is conceptually problematic, and currently contested as previously discussed (Nolan, 2013). Superannuation policy, for instance, in Australia is arguably social investment in financial capital accumulation (i.e., part of the ‘piggy-bank’ function of the welfare state alongside the ‘Robin Hood’ protective welfare function that redistributes resources within society). The minimum-income protection floor for pensioners was raised to 28 per cent of average weekly male earnings, following the Harmer Review (2009) of the pensions system, and the Superannuation Guarantee rate was set to increase from 9 per cent in 2012 to 12 per cent by 2018, following the Cooper Review (2010), benefiting 8.4 million Australians. The Australian health service was also strengthened under Labor, another key social democratic reform, and Labor introduced DisabilityCare Australia to support people with significant and permanent disability. In many ways, the Labor government was an ambitious welfare-state builder but, in other respects, the Australian system of welfare was arguably attempting to catch up with social welfare systems found in many of the other developed economies. Importantly, Figure 4 does show a notable rise in the level of public expenditure as Labor strengthened health, education, pensions and disability services.

## Social investment and equality

In this section, we move to consider in more detail the social security policy framework in Australia and some of the distributional impacts and consequences of Labor's social investment and inclusion strategy. We are particularly interested in social protection and out-of-work benefits; and the long-running trends in poverty and inequality, thus placing Australian trends within a comparative context. We also consider gender inequalities and Indigenous peoples’ disadvantage. Proponents of social investment maintain that a secure welfare safety net of minimum income support is an imperative but, with the focus on productive investment and activation in the Australian context, it is far from self-evident that adequate social protection has been secured during the latest round of social democratic reforms. Most importantly, unemployment risks themselves are not evenly distributed across society, and risk is increasingly concentrated at the bottom of the socio-economic class structure. Low-skilled workers, for example, face a different and altogether higher risk of unemployment than skilled workers and middle-class professionals.

**Figure 5.**
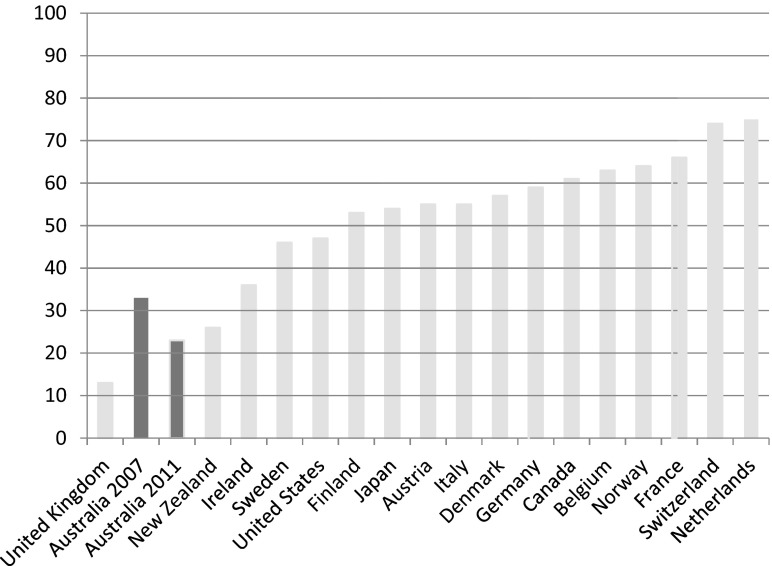
Unemployment benefit replacement rates for adults in the advanced economies (% of average wage levels in 2011, plus 2007 for Australia) *Source:* OECD Employment and Labour Markets Statistics (DOI: 10.1787/lfs-data-en).

**Figure 6.**
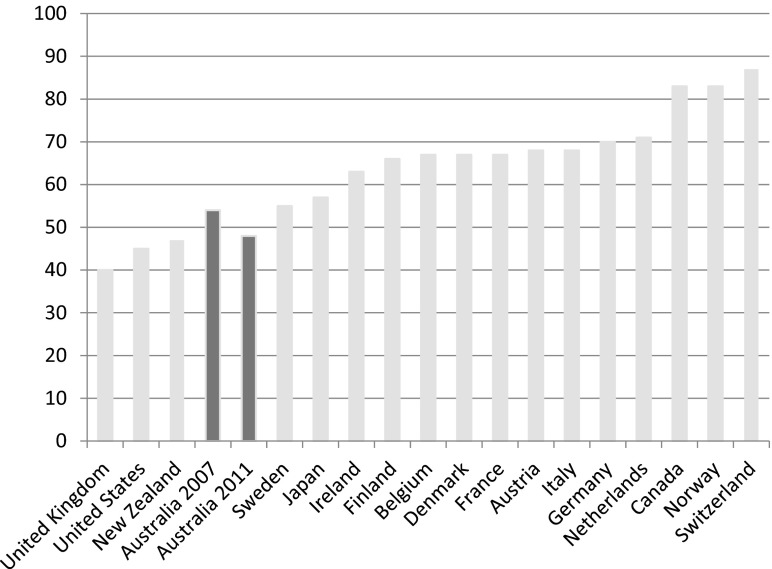
Unemployment benefit replacement rates for a single parent with two children in the advanced economies (% of average wage levels in 2011, plus 2007 for Australia) *Source:* OECD Employment and Labour Markets Statistics (DOI: 10.1787/lfs-data-en).

**Figure 7.**
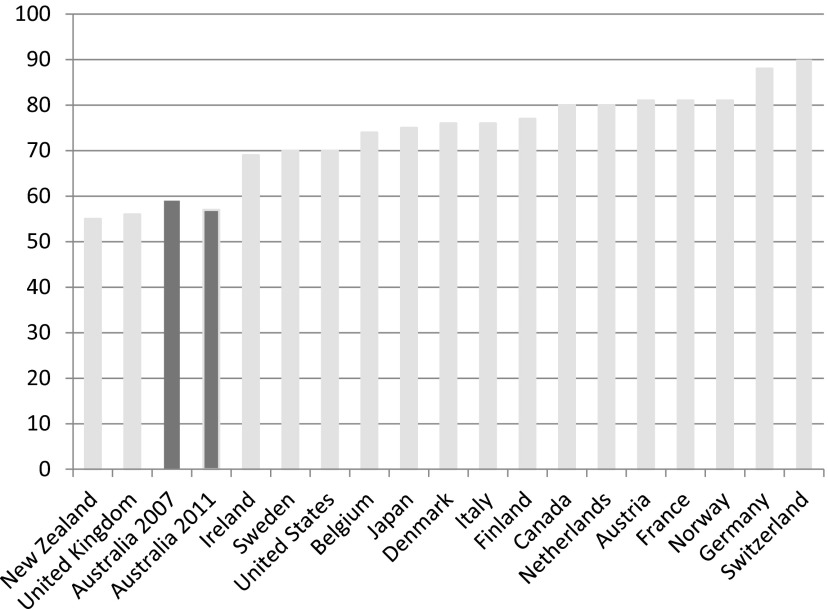
Unemployment benefit replacement rates for a couple with two children in the advanced economies (% of average wage levels in 2011, plus 2007 for Australia) *Source:* OECD Employment and Labour Markets Statistics (DOI: 10.1787/lfs-data-en).

There is good evidence now emerging that suggests investment in human capital may have been accompanied by greater constraints imposed on Australia's system of income support. So, while minimum wage schemes and working tax credits helped to ensure that work paid, wage replacement rates fell in real terms by comparison with average earnings. In 2005, for example, unemployment benefit replacement rates for an adult stood at 33 per cent of average wages compared to just 23 per cent in 2011 – a 10 per cent fall against average earnings. Benefit levels in Australia continue to be amongst the lowest in the Western world. Thus, Australians appear to accept the risk of a relatively low standard of living and poverty if they are unable to work or become unemployed. Benefit replacement rates, paid in the initial phase of unemployment, are low compared to other advanced economies. For an unemployed adult, benefit replacement rates amount to about one-fifth of average wages; only unemployed adults in the UK receive less (Figure 5). Wage replacement rates for single parents and couples with children in Australia are better, but they remain relatively low in comparison to the other OECD nations (Figures 6 and 7).

In many ways, Labor's approach to social investment and inclusion remained distinctly neoliberal or ‘light’, characteristic of the ‘Work First’ approach (any job is better than inactivity). Labor continued to develop the workfare-orientated strategy that relies on setting very strong work incentives, largely at the expense of income support allowances – already low by international standards. Benefit conditionality increased under Labor and spending on ALMPs, again relatively low by international standards, remained unchanged over the 2007–13 period.

Investment in the social security system for unemployed workers and vulnerable citizens living on out-of-work benefits was not accorded a priority. Liberal welfare states have long assumed that individuals prefer not to work if minimum wages are undermined by decent social security benefits (i.e., ‘moral hazard’); in the Australasian context this has particular resonance as work incentives have long taken priority over any meaningful standard of adequacy in social policy (Deeming, 2013a). During Labor's term in office, social policy scholars (e.g., Soldatic and Pini, 2012) continued to call into question the adequacy of social security benefits for those not in paid work. Labor, however, was essentially content to preserve Australia's system of redistribution and arguably accepted the political limits and constraints imposed by the low-tax approach to social democracy (Deeming, 2013b; Wilson, 2013). In Australia, the burden of tax on waged labour is low by international standards, only Switzerland and New Zealand appear to have lower tax rates than Australia. Indeed, a major independent review of taxation policy in 2009 (Henry Review, 2009) argued that the country's tax and transfer system, which has long favoured market freedom and individual opportunity, had served Australia well, and the Labor government accepted this verdict.

**Figure 8.**
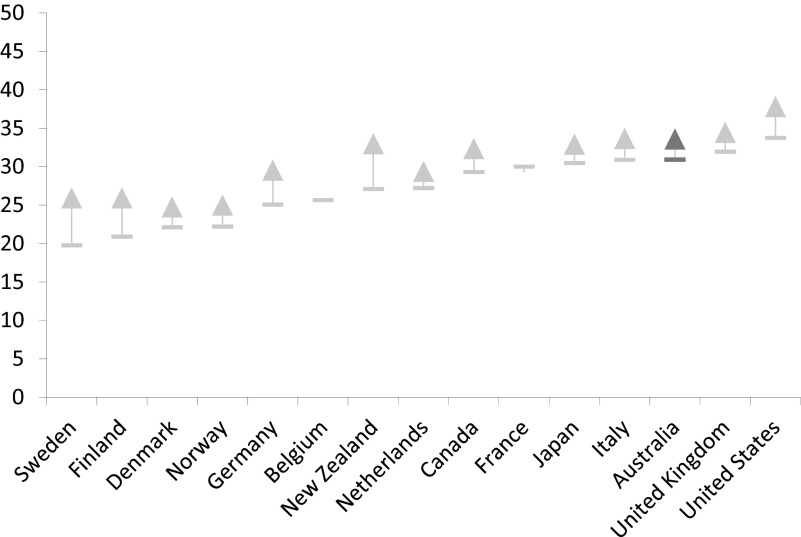
Income inequality in the advanced economies, mid-1980s to the late 2000s (Gini coefficients) *Note:* Arrows indicate the change and direction of income inequality. *Source:* OECD Income Distribution and Poverty Database (IDD in OECD.Stat).

These reforms go to the heart of the question of whether growth in the Australian economy has been inclusive. A strong economy and low levels of unemployment have lifted many but not all boats; a small minority of the Australian population has remained jobless. Most of the gains from economic growth were highly concentrated at the top end of the income distribution, and as a result we find relative poverty and inequality has risen in recent times. Figure 8, for example, shows widening inequality since the mid-1980s, increasing by 1.6 percentage points on the Gini index.^2^

**Figure 9.**
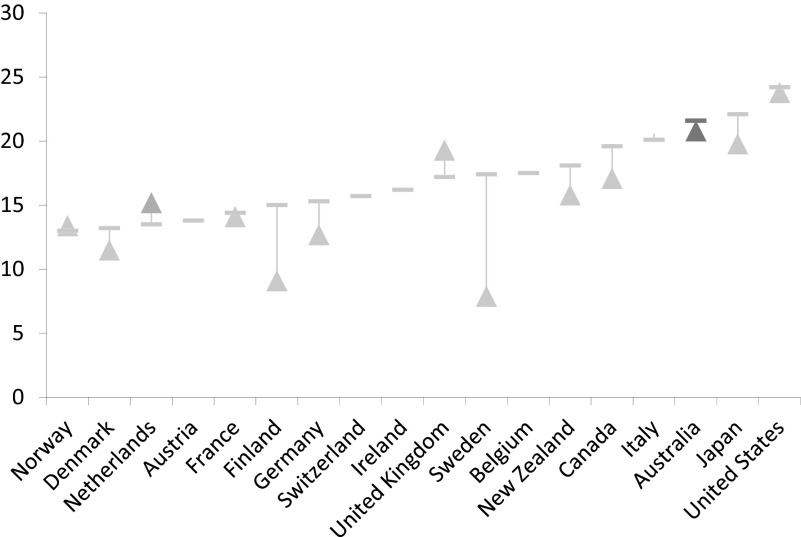
Poverty rates in the advanced economies, mid-1990s to the late 2000s (60% of median income) *Note:* Arrows indicate the change and direction of income poverty. *Source:* OECD Income Distribution Database (IDD in OECD.Stat).

Poverty in Australia has also increased since the mid-1990s, by one percentage point. Over a fifth (22 per cent) of Australians now live with disposable household income below the 60 per cent poverty threshold. As shown in Figure 9, this puts Australia above most of the other advanced economies on this measure of hardship. And while some of the latest evidence on social exclusion in Australia points to a decline in levels of exclusion overall as a result of Labor's policies, nevertheless prospects for some of Australia's most excluded citizens did not improve over the 2007–13 period (Saunders, 2013). So, while the size of the group in this position appears to have shrunk, its distance from the mainstream has undoubtedly grown.

Progress on tackling Indigenous peoples’ disadvantage remained slow despite a range of policy initiatives such as ‘Closing the Gap’, designed to create a more inclusive Australia. The unemployment rate for people of Aboriginal and Torres Strait Islander origin in Australia, at 20 per cent, is over three times the national average. Indigenous Australians tend to live in areas of socio-economic disadvantage and experience a gap in life expectancy at birth estimated to be 11.3 years for males and 9.5 years for females, compared to total males and females. Less than one-third of young Indigenous Australians (aged between seventeen and eighteen years) and only about half of young people from low socio-economic backgrounds obtain the school leaving qualification (Whiteford, 2013). Youth unemployment continues to be a concern and gender inequalities stubbornly persist in Australian society. Australia is a long way from gender pay parity – a key measure of fairness – despite the policy of ‘equal pay for equal work’. From 2010, working parents were given the right to request flexible work arrangements and working times. State policy clearly matters but state-level provision is shaped in important ways at the organisational level and within the workplace. Women are much more ‘flexible’ workers than men. In Australia, 70 per cent of part-time employment is female (so in practice Australia now has mainly one (male) and a half (female) ‘bread-winner’ families). Part-time work and fixed-term contracts help to explain the inferior labour market position of women, who are disproportionately responsible for care work (Craig and Mullan, 2011). The re-engineering of welfare policy in the name of social investment by the Labor government arguably struggled to tackle some of the deeply entrenched gender, cultural and ethnic inequalities. Drawing together the continuities and breaks in ‘social investment’ policy, as implemented by the Australian Labor Party, we find improvements in living standards for most Australians, but continued economic growth has not been inclusive for all. Persistent poverty, multiple and entrenched social disadvantage and growing social inequality continue to trouble Australian society (Leigh, 2013).

In the European context, social policy scholars warn that the shifting emphasis of social policy towards social investment in human capital policies and labour market integration may well come at the expense of social protection and inclusion for all. Certainly there is growing evidence for this in Europe (Cantillon and Van Lancker, 2013; Van Kersbergen and Hemerijck, 2012), and this must be the concern within the Australian context. Social security against the adverse effects of the labour market is still of value to society and the citizens who are protected by social policy. Thus, employment ‘flexibility’ and traditional social protection, in the form of a secure safety net that provides workers with an adequate standard of living in the event of unemployment is a precondition for an effective ‘heavy’ social investment strategy that promotes equality and protects against poverty in the event of unemployment.

Early on in its term, the Labor government appears to have acknowledged that it faced heavy constraints and inevitable limits were placed on its strategy of ‘social investment’. Thus, the government posed the relevant policy question: ‘Does Australia's level of social expenditure underwrite the social investments necessary for an inclusive society?’ The answer, it would seem, turns out to be decisive:

*Even after allowing for difficulties in harmonising different accounting systems, the conclusion is inevitable that Australia stands near the bottom of the list of relative social expenditures* (Australian Government, 2009: 81, emphasis added).

Here Labor seems to have accepted that the necessary platform for social investment in Australia was ‘light’ at best; essentially concluding that Australia's overall level of social expenditure was probably unlikely to be sufficient to underwrite the level of ‘heavy’ social investment needed for a more inclusive society. Even after allowing for difficulties in harmonising international data, as the government suggests, it is clear Australia's social investment strategy looks different from many other comparable European countries in a number of important respects. Investment in ALMPs remains low, as do social expenditure levels more generally, although health and education have benefited from additional investment. Out-of-work benefits, already low by international standards, lost value against earnings. In this context, Labor's legacy appears to have been the recognition that greater investment was needed, but the accompanying revenue-raising strategy was out of reach. Hence, the low tax road towards ‘social investment’.

## Conclusions

In many ways, the Australian experience reflects the general move away from the neoliberal model of economy and society, that dominated the late twentieth century, and the desires of Australian social democrats, who returned to the ‘social investment’ paradigm that seeks inclusive growth (this time for all Australians) and once again regards social policy as economic investment.

In a number of important respects, however, the Australian approach (and that of other liberal market economies) differs from the social investment strategies being pursed in some European contexts (Morel *et al.*, 2012). In the liberal state of Australia, as we have shown, there has been a decisive move to strengthen the social policy framework for ‘hardworking families’ – ‘the battlers’ against the ‘dole bludgers’ – as rhetorical incarnations of economic struggle (or the ‘strivers’ verses the ‘skivers’ in the British context).^3^ In the battle over the political middle ground, more affluent middle-class families have been brought into the social security system. Dual-earner families on low-to-middle incomes now receive ‘welfare-in-work’. Under Labor, social investment was orientated towards meeting their needs and protecting their living standards – although greater efforts are required to tackle gender inequalities in the labour market and caring roles in the home. Nevertheless, the range of initiatives implemented by Labor can be seen as forging a new *de facto* ‘contract’ whereby the state has now absorbed greater responsibility for complementing the ‘market family wage’, at least for working families. From this perspective, there has been a complete transformation of the Australian welfare state; the dual-earner family-centred mode of household welfare production is fast replacing the vacuum left by the ‘male bread-winner’ model. Here we might also wish to draw attention to the continued role of politics in the policy process, determining social policy outcomes. The old ‘male breadwinner/female home-maker’ family model of welfare grew out of the democratic class struggle and, in turn, it is a new politics that has shaped, if not constrained, the path of ‘social investment’ in Australia (Deeming, 2013b).

Labor's defining achievement has been to recreate a state of investment for ‘working families’, and, in the social democratic tradition, one that has benefited working families at the lower end of the income distribution, which also protects their living standards in retirement, and all Australians in the event of poor health and/or disability. In other ways, Labor's ‘light’ approach to social investment strengthened the policy of ‘workfarism’ it had inherited from the Howard-led Liberal–National coalition government (1996–2007), taking a more punitive stance towards social assistance recipients in an effort to move non-working citizens off welfare benefits and into paid employment. A more fully engaged ‘heavy’ social investment scenario that offers better insurance against the risk of unemployment is costly and has never carried much appeal with the Australian electorate. Under the Labor government, social protection lost ground to rising living standards in the working population, and social inequality has in many respects become more visible.

The article therefore exposes some of the tensions inherent in the pursuit of ‘social investment’ and inclusion strategies by leftist parties within the ‘neoliberal state’ (Plant, 2012). Social democrats in Australia face particular challenges where political institutions facilitate the pursuit of private ends, rather than defined political goals like ‘social investment’ for a more just society. At the same time, we also find the Nordic welfare states in the midst of transformation (Kananen, 2014). All eyes are now on the Nordic countries to see if ‘heavy investment’ which has produced ‘high efficiency’ in the capitalist market economy with ‘high equity’ in the distribution of resources and life chances in society can be maintained in the face of global forces and pressures from the new international economic order.

## Notes

1 The notion that unemployment benefits are ‘passive’ can be problematic. Out-of-work benefits help to support livelihoods and aid workers in their search for new employment.

2 The Gini index measures the extent to which the income distribution deviates from a perfectly equal distribution: 0 represents perfect equality and 100 implies perfect inequality.

3 Regrettably, such misleading rhetoric being used by politicians seriously risks undermining trust and social welfare services still further.

## References

[ref001] Australian Department of the Premier and Cabinet (2005), A Third Wave of National Reform: A New National Reform Initiative for COAG – The Proposals of the Victorian Premier, Melbourne: Australian Government Publishing Service.

[ref002] Australian Government (2009), Compendium of Social Inclusion Indicators: How's Australia Faring?, Barton ACT: Commonwealth of Australia.

[ref003] Beblavý M., Thum A.-E. and Veselkova M. (2013), ‘Education and social protection policies in OECD countries: social stratification and policy intervention’, Journal of European Social Policy, 23: 5, 487–503.

[ref004] Belchamber G. (2013), ‘To fix a flaw and fix the floor: unemployment insurance for Australia’, in P. Smyth and J. Buchanan (eds.), Inclusive Growth in Australia: Social Policy as Economic Investment, Sydney: Allen & Unwin, pp. 193–204.

[ref005] Blaxland M. (2013), ‘Street-level interpellation: how government addresses mothers claiming income support’, Journal of Social Policy, 42: 4, 783–97.

[ref006] Bonoli G. (2009), ‘Varieties of social investment in labour market policy’, in N. Morel, B. Palier and J. Palme (eds.), What Future for Social Investment?, Stockholm: Institute for Futures Studies, pp. 55–66.

[ref007] Bonoli G. (2012), ‘Active labour market policy and social investment: a changing relationship’, in N. Morel, B. Palier and J. Palme (eds.), Towards a Social Investment Welfare State? Ideas, Policies and Challenges, Bristol: Policy Press, pp. 181–204.

[ref008] Brumby J. (2013), Reform or Perish − A Report Card on Five Years of National Reform, 6 November, National Press Club, Canberra ACT, http://www.coagreformcouncil.gov.au/media/speeches/2013-11-06 (accessed October 2014).

[ref009] Burke G. (2013), ‘Skills for growth and inclusion’, in P. Smyth and J. Buchanan (eds.), Inclusive Growth in Australia: Social Policy as Economic Investment, Sydney: Allen & Unwin, pp. 132–54.

[ref010] Cantillon B. and Van Lancker W. (2013), ‘Three shortcomings of the social investment perspective’, Social Policy and Society, 12: 4, 553–64.

[ref010a] Cooper Review (2010), Super System Review Final Report, Barton, ACT: Commonwealth of Australia.

[ref011] Craig L. and Mullan K. (2011), ‘How mothers and fathers share childcare: a cross-national time-use comparison’, American Sociological Review, 76: 6, 834–61.

[ref012] Deeming C. (2013a), ‘The working class and welfare: Francis G. Castles on the political development of the welfare state in Australia and New Zealand thirty years on’, Social Policy and Administration, 47: 6, 668–91. 24436502 10.1111/spol.12037 PMC3886295

[ref013] Deeming C. (2013b), ‘Social democracy and social policy in neoliberal times’, *Journal of Sociology*, 50 (4): 577–600. 10.1177/1440783313492240 PMC4246053 25473376

[ref014] Esping-Andersen G. (2001), ‘A welfare state for the 21st century’, in A. Giddens (ed.), The Global Third Way Debate, Cambridge: Polity, pp. 134–56.

[ref015] Esping-Andersen G., Gallie D., Hemerijck A. and Myles J. (eds.) (2002), Why We Need a New Welfare State, Oxford: Oxford University Press.

[ref016] European Commission (2000), Social Policy Agenda, COM(2000) 379 final, Communication from the Commission, Brussels: European Commission.

[ref017] European Commission (2013), Towards Social Investment for Growth and Cohesion, COM(2013) 83 final, Communication from the Commission, Brussels: European Commission.

[ref018a] Giddens A. (1998), The Third Way, Cambridge: Polity Press.

[ref018] Hall P. A. (ed.) (1989), The Political Power of Economic Ideas: Keynesianism Across Nations, New Jersey, NJ: Princeton University Press.

[ref019] Hall P. A. (1993), ‘Policy paradigms, social learning, and the state: the case of economic policymaking in Britain’, Comparative Politics, 25: 3, 275–96.

[ref018b] Harmer Review (2009), Pension Review Report, Canberra ACT: Commonwealth of Australia.

[ref020] Hemerijck A. (2012), ‘The political economy of social investment’, in L. Burroni, M. Keune and G. Meardi (eds.), Economy and Society in Europe: A Relationship in Crisis, Cheltenham: Edward Elgar, pp. 40–60.

[ref020a] Henry Review (2009), Australia's Future Tax System: Report to the Treasurer, Part One: Overview, Barton ACT: Commonwealth of Australia.

[ref021] Jenson J. (2010), ‘Diffusing ideas for after neoliberalism: the social investment perspective in Europe and Latin America’, Global Social Policy, 10: 1, 59–84.

[ref022] Jenson J. (2012), ‘Redesigning citizenship regimes after neoliberalism: moving towards social investment’, in N. Morel, B. Palier and J. Palme (eds.), Towards a Social Investment Welfare State? Ideas, Policies and Challenges, Bristol: Policy Press, pp. 61–87.

[ref023] Kananen J. (2014), The Nordic Welfare State in Three Eras: From Emancipation to Discipline, Surrey: Ashgate.

[ref023a] Kvist J. (2014), A framework for social investment strategies: Integrating generational, life course and gender perspectives in the EU social investment strategy, *Comparative European Politics*, DOI: 10.1057/cep.2014.45 published online ahead of print, http://dx.doi.org/10.1057/cep.2014.45 (accessed October 2014).

[ref024] Leigh A. (2013), Battlers and Billionaires: The Story of Inequality in Australia, Collingwood: Redback.

[ref025] Midgley J. (1999), ‘Growth, redistribution, and welfare: toward social investment’, Social Service Review, 73: 1, 3–21.

[ref026] Morel N., Palier B. and Palme J. (2012), ‘Social investment: a paradigm in search of a new economic model and political mobilisation’, in N. Morel, B. Palier and J. Palme (eds.), Towards a Social Investment Welfare State? Ideas, Policies and Challenges, Bristol: Policy Press, pp. 353–76.

[ref027] Nolan B. (2013), ‘What use is “social investment”?’, Journal of European Social Policy, 23: 5, 459–68.

[ref028] Organisation for Economic Cooperation and Development (OECD) (2010), Economic Policy Reforms 2010: Going for Growth, Paris: OECD.

[ref029] Organisation for Economic Cooperation and Development (OECD) (2014), Changing the Conversation on Growth: Going Inclusive, Paris: OECD.

[ref030] Perkins D., Nelms L. and Smyth P. (2005), ‘Beyond neo-liberalism: the social investment state?’, Just Policy: A Journal of Australian Social Policy, 38: 34–41.

[ref031] Plant R. (2012), The Neo-liberal State, Oxford: Oxford University Press.

[ref032] Saunders P. (2013), ‘Reflections on the concept of social exclusion and the Australian social inclusion agenda’, Social Policy and Administration, 47: 6, 692–708.

[ref033] Saunders P. and Deeming C. (2011), ‘The impact of the crisis on Australian social security policy in historical perspective’, Social Policy and Administration, 45: 4, 371–88.

[ref033a] Sen A. (2005), ‘Human Rights and Capabilities’, Journal of Human Development, 6: 151–66.

[ref034] Smyth P. (2008), ‘Closing the gap? The role of wage, welfare and industry policy in promoting social inclusion’, Journal of Industrial Relations, 50: 4, 647–63.

[ref035] Smyth P. and Buchanan J. (eds.) (2013), Inclusive Growth in Australia: Social Policy as Economic Investment, Sydney: Allen & Unwin.

[ref036] Soldatic K. and Pini B. (2012), ‘Continuity or change? Disability policy and the Rudd government’, Social Policy and Society, 11: 2, 183–96.

[ref037] Tilton T. (1993), ‘The role of ideology in social democratic politics’, in K. Misgeld, K. Molin and K. Amark (eds.), Creating Social Democracy: A Century of the Social Democratic Labor Party in Sweden, University Park Pennsylvania: Pennsylvania State University Press, pp. 409–27.

[ref038] Van Kersbergen K. and A. Hemerijck (2012), ‘Two decades of change in Europe: the emergence of the social investment state’, Journal of Social Policy, 41: 3, 475–92.

[ref039] Whiteford P. (2013), ‘Australia: Inequality and Prosperity and Their Impacts in a Radical Welfare State’, in B. Nolan, W. Salverda, D. Checchi, I. Marx, A. McKnight, I. G. Tóth and H. G. van de Werfhorst (eds.), Changing Inequalities and Societal Impacts in Rich Countries: Thirty Countries' Experiences, Oxford: Oxford University Press, 48–70.

[ref040] Wilson S. (2013), ‘The limits of low-tax social democracy? Welfare, tax and fiscal dilemmas for labor in government’, Australian Journal of Political Science, 48: 3, 286–306.

